# The performance of a flapping foil for a self-propelled fishlike body

**DOI:** 10.1038/s41598-021-01730-4

**Published:** 2021-11-16

**Authors:** Damiano Paniccia, Luca Padovani, Giorgio Graziani, Renzo Piva

**Affiliations:** Department of Mechanical and Aerospace Engineering, University of Rome “La Sapienza”, Rome, Italy

**Keywords:** Fluid dynamics, Mechanical engineering, Biomimetics

## Abstract

Several fish species propel by oscillating the tail, while the remaining part of the body essentially contributes to the overall drag. Since in this case thrust and drag are in a way separable, most attention was focused on the study of propulsive efficiency for flapping foils under a prescribed stream. We claim here that the swimming performance should be evaluated, as for undulating fish whose drag and thrust are severely entangled, by turning to self-propelled locomotion to find the proper speed and the cost of transport for a given fishlike body. As a major finding, the minimum value of this quantity corresponds to a locomotion speed in a range markedly different from the one associated with the optimal efficiency of the propulsor. A large value of the feathering parameter characterizes the minimum cost of transport while the optimal efficiency is related to a large effective angle of attack. We adopt here a simple two-dimensional model for both inviscid and viscous flows to proof the above statements in the case of self-propelled axial swimming. We believe that such an easy approach gives a way for a direct extension to fully free swimming and to real-life configurations.

## Introduction

**Figure 1:**
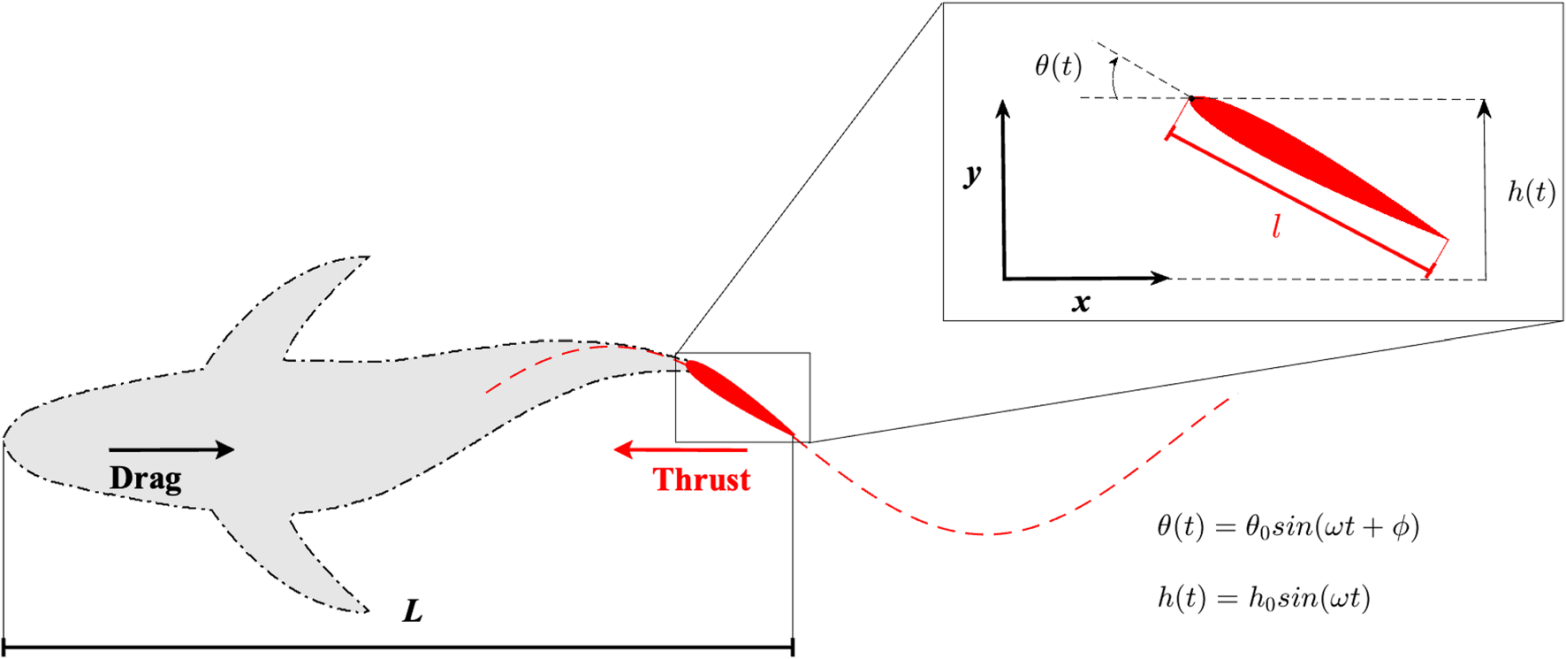
A cartoon for the virtual body (gray) and the tail propulsor (red) with a sketch of the exchanged forces and the oscillatory trajectory of the tail pivot point. The details of the flapping motion are reported in the inset. See also the animation of the swimming fishlike model and the related vortex wake in the Supplementary Video online.

The self-propulsion of certain fishes may be reduced to the study of the oscillatory motion of the caudal fin. For instance, a tunniform swimmer uses the tail to generate most of the propulsive force, while the anterior part of the body provides essentially a viscous resistance. In these cases since it is possible, as a first approximation, to separate drag and thrust, in the past it was considered more convenient to study the tail as an isolated flapping foil, i.e. with a combined heave and pitch motion. Actually, most of the attention was paid to the study of a flapping foil under a uniform incoming stream to evaluate the fluid-induced thrust which is able to counteract the unavoidable body resistance. Hence, the Froude efficiency $\eta = T U/P$ (*T* thrust, *P* input power and *U* inflow velocity) was used as a measure for the performance of the propulsion system, repeatedly analyzed in many contributions either analytical^1,2^, numerical^3,4^ or experimental^5,6^. However, the primary interest remains the evaluation of self-propelled swimming properties like the locomotion speed and the energy consumption hence we propose here to recover the approach properly adopted when the whole body is cooperating for the generation of the required thrust. This is the case of undulatory swimming, where a wave travelling from head to tail is involving a significant part of the body consistently with the fish’s shape and swimming style (e.g. anguilliform, carangiform, etc.). For these reasons, a clear identification of the propulsive efficiency is prevented^7–9^ and, after a few initial studies with a prescribed stream^10,11^, the self-propelled locomotion velocity was obtained by leaving the fish completely free to swim according to the forces exchanged with the surrounding fluid^12–15^. When at steady state thrust and drag balance exactly, the Froude efficiency loses its meaning and a proper concept like the cost of transport $COT = P/U$, or its inverse as introduced by von Kármán and Gabrielli^16^, should be considered instead^17,18^. By proceeding in an analogous way for oscillatory swimming, we intend to investigate the axial self-propulsion of a flapping foil pushing a fishlike body which, in a way passive with respect to the thrust, may be approximated by defining only its mass and its resistance, i.e. a virtual body as proposed by Akoz^19^. These assumptions, due to the known resistance and to the axial motion of the virtual body, allow for the evaluation of the cost of transport as a measure for the self-propelled swimming performance, but also for a clear-cut evaluation of the Froude efficiency providing an easy comparison between the optimal conditions for the two performance measures. By following other suggestions from the seminal work of Schultz and Webb^20^ and later by Gazzola et al.^21^, we prefer to concentrate our attention on two dimensional simulations to achieve a sharp understanding of the complex phenomena just described. A cartoon for the virtual body and its tail propulsor with a sketch representing the exchanged forces and the oscillatory trajectory for the tail pivot point is reported in Fig. 1. The animation reported in the Supplementary Video online gives a first glance insight of the swimming fishlike model and of the related vortex wake.

## Results and discussion

### A test for zero resistance

As a preliminary step, we like to consider the self-propelled axial motion of a virtual body having zero resistance^22–25^, a sort of ideal case, to highlight in the most simple and neat way the possible analogies with the undulatory swimming mode. For instance, we like to understand if an asymptotic locomotion velocity occurs also for a flapping foil and how to find a good approximation of its value. This is feasible if the pitch motion about the leading edge, with an angular frequency $\omega$ and a small amplitude $\theta _0$, anticipates the heave motion, with amplitude $h_0$, by a phase angle $\phi = \pi /2$. With these assumptions, we may express the flapping motion of a foil with chord *l* as

1 $$\begin{aligned} y(x,t) = h_0 \sin (\omega t) - x \sin (\theta (t)) \approx h_0 \sin (\omega t) - \theta _0 x \cos (\omega t) \quad 0 \le x \le l \end{aligned}$$

which may be assimilated to an undulatory motion of amplitude *A* and wavenumber *k*.

When the wavelength $\lambda = 2\pi /k \gg l$, this motion may be expressed as

2 $$\begin{aligned} y(x,t) = A \sin (\omega t - k x) \approx A \sin (\omega t) - A k x \cos (\omega t) \quad 0 \le x \le l \end{aligned}$$

and, by identifying the single terms of (1) and (2), we obtain for the phase velocity

3 $$\begin{aligned} c = \frac{\omega }{k} \approx \omega \frac{h_0}{\theta _0} \end{aligned}$$

Intuitively, if $\lambda \gg l$, the foil itself acts as a small portion of the wave whose undulating motion is perceived, instantaneously, as a local oscillation given by the heave and pitch motions.

**Figure 2:**
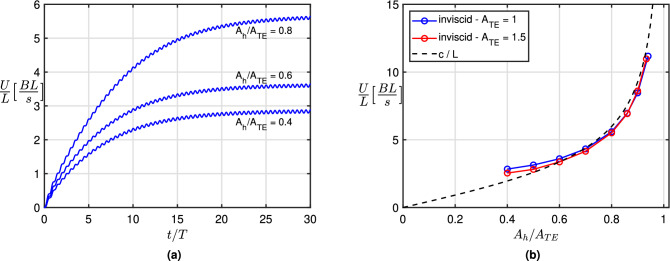
(**a**) Time history of the locomotion speed for $A_{TE} = 1$ and three different values of $A_h/A_{TE}$. (2b) Mean steady state swimming velocity *U*/*L* and phase velocity *c*/*L* (dashed line) against $A_h/A_{TE}$ for different peak-to-peak trailing edge oscillation amplitudes ($A_{TE} = 1$ and 1.5). Inviscid numerical results for zero resistance of the virtual body.

**Figure 3:**
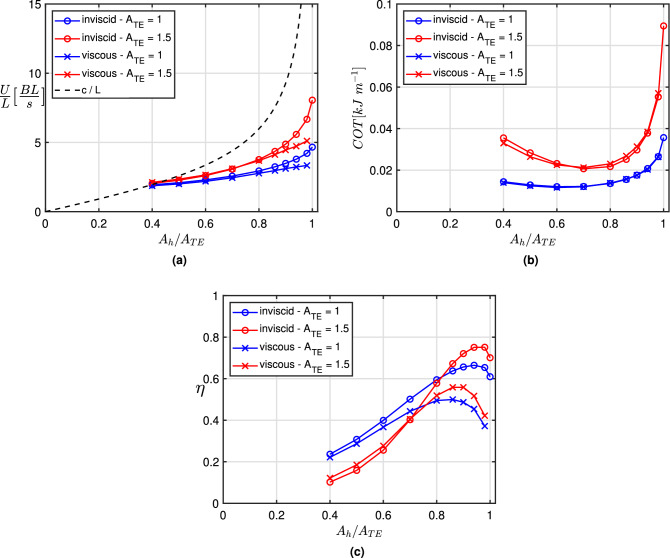
(**a**) Mean steady state swimming velocity *U*/*L* and phase velocity *c*/*L* (dashed line), (**b**) cost of transport of the whole body and (**c**) efficiency of the propulsor against $A_h/A_{TE}$ for different peak-to-peak trailing edge oscillation amplitudes ($A_{TE} = 1$ and 1.5). Viscous and inviscid numerical solutions for a prescribed virtual body resistance.

Consistently with the assumption of small $\theta _0$, the above analogy becomes more and more accurate as the wavelength $\lambda$ is greater than the tail length. This phase velocity gives us the opportunity to recall the proportional-feathering parameter $\Theta = \theta _0 U / \omega h_0$, as ingeniously suggested by Lighthill^26^, to qualify the propulsive performance of flapping foils. Actually, the expression for $\Theta$ results to be the ratio between the locomotion velocity *U* and the phase velocity *c* given by (3), to match the concept of slip velocity usually adopted in undulatory swimming^27^.

For the analysis of the numerical results in the self-propelled case, since both the Strouhal number $St = \omega lA_{TE}/(2\pi U)$ and the reduced frequency $k_r = \omega l/U$ contain the forward velocity *U* which is part of the solution, we should select new parameters strictly based on the assigned data. To this purpose, we introduce the non-dimensional trailing edge peak-to-peak oscillation amplitude $A_{TE}$ in terms of the foil chord *l* and the pure heave non-dimensional peak-to-peak amplitude defined as $A_h = 2 h_0/l$. For a given value $A_{TE}$, which for small values of $\theta _0$ may be approximated by $\sqrt{(2\theta _0)^2+A_h^2}$, the ratio $A_{h}/A_{TE}$ is the parameter that we are going to use to analyze the results. It represents the fraction of the trailing edge amplitude due to heave, so as $A_h/A_{TE} = 0$ for pure pitch and 1 for pure heave.

The time history of the forward locomotion velocity obtained by a standard inviscid numerical procedure for the zero resistance virtual body is reported in fig.2a for $A_{TE} = 1$ and three different values of $A_h/A_{TE}$. From the figure we may appreciate how the acceleration during the transient is increasing with the heave amplitude to reach anyhow, even in the absence of a viscous resistance, an asymptotic velocity which is going to infinity for pure heave. We like to notice that the forward velocity oscillations appearing in the figure are very small and their global effect on swimming performance is quite negligible as assumed in a previous work on recoil motions^28^ and confirmed by a present simulation reported in the Supplementary Material (see also^17^ and^29^). The mean forward velocity at the steady state, for $A_{TE}$ equal to 1 and 1.5, is plotted against $A_h/A_{TE}$ in Fig. 2b together with the phase velocity *c* which is only a function of the ratio $A_{h}/A_{TE}$. Let us remark that the selected values of $A_{TE}$ correspond nearly to $0.15-0.2$ in terms of the ratio between the tail-beat amplitude and the body length *L*, as frequently observed in nature^30^. As anticipated above, for small pitch angles, i.e. for $A_h/A_{TE}$ going to one, the prediction of the asymptotic velocity equal to *c* is confirmed by the numerical results which also show how the locomotion speed is practically independent of the trailing edge amplitude $A_{TE}$, as for undulatory swimming in the specific case of inviscid flows^31^. For zero resistance, the Froude efficiency continuously decreases up to a null value at steady state where the net thrust is going to vanish. At the same time, the cost of transport is decreasing towards steady state but it reaches an asymptotic finite value resulting extremely low due to a reduced expended energy and a very large locomotion speed in absence of viscous resistance. This expected behaviour is properly modified when introducing a non zero virtual body resistance leading to intermediate values of both *COT* and $\eta$ with respect to the above extreme conditions. At steady state the efficiency is not zero anymore since the thrust reaches a finite value able to counterbalance the imposed drag and the cost of transport gains a value consistent with a reduced locomotion speed together with an increase of the expended power. As shown by the numerical results in the following section, it is easy to understand the primary role of the body resistance to qualify the overall performance of the swimming fish.

### The role of the body resistance

As anticipated before, the concept of virtual body introduced to estimate the performance of the fishlike body, requires an assumption for the drag coefficient $C_D$ as close as possible to expected real values which may be selected from experimental evidence^32^.

**Figure 4:**
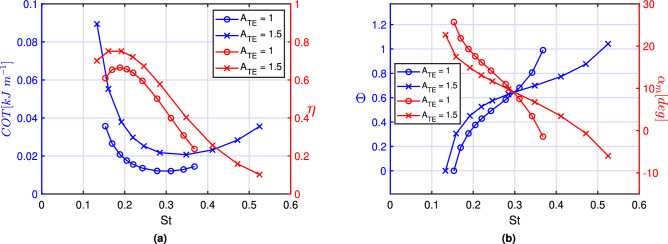
(**a**) Cost of transport of the whole body (blue) and efficiency of the propulsor (red) as function of the Strouhal number *St*. (**b**) Feathering parameter $\Theta$ (blue) and maximum angle of attack $\alpha _{m}$ (red) for the inviscid case as function of the Strouhal number. Comparison between $A_{TE} = 1$ and 1.5 for the inviscid case.

The mean forward velocity is shown in Fig. 3a for viscous and inviscid flows together with the phase velocity *c*. We may notice a general reduction of the velocity values with respect to Fig. 2b clearly due to the extra virtual body resistance and their dependence on the peak-to-peak trailing edge amplitude $A_{TE}$, as expected in the presence of viscous resistance^33^. A contained difference is appreciable when comparing viscous and inviscid results, in particular for larger values of $A_h/A_{TE}$, essentially related to the viscous resistance of the propulsor which increases with the locomotion speed to give a sensible difference between the two approaches^34^. The whole body performance given by the cost of transport is shown in Fig. 3b with a very satisfactory agreement between viscous and inviscid results. A clear evidence of the classical U-shaped form for the COT curves^35,36^ is obtained and a minimum value appears in a quite small range about $A_h/A_{TE} = 0.7$. The presence of a virtual body allows also for the calculation of the propulsor efficiency since the thrust, balancing the known drag at cruising speed, is now available^37,38^. Interestingly, the range where we find the maximum efficiency of the propulsor, reported in Fig. 3c, is clearly different from the one where the minimum *COT* for the whole body occurs. Specifically, the range corresponding to the maximum value of the efficiency $\eta$ is found for larger values of $A_h/A_{TE}$. Let us mention that other authors, by making different choices, may obtain different results which however are perfectly compatible with the present ones. For instance, Akoz et al.^19^, by forcing a constant self-propelled locomotion speed for a defined body via a change of frequency, interestingly find the cost of transport as the inverse of the propulsor efficiency. Instead, if a constant speed is prescribed without caring for the self-propelled conditions consistent with a given body resistance^3,4,6^, the attention is only focused on the generic properties of the propulsor as clearly underlined by Anderson et al.^5^.

For the sake of completeness, we illustrate in Fig. 4a the values of cost of transport and efficiency also in terms of the more commonly used Strouhal number that was previously set apart for its dependence on the unknown locomotion velocity. The figure confirms the results previously discussed about the substantial difference of the optimal ranges for the two performance measures. Within this context it is worth stressing how the optimal values of *COT* and $\eta$ are related to a couple of very significant parameters i.e. the proportional-feathering $\Theta$ and the maximum effective angle of attack $\alpha _m$. Following Anderson et al.^5^, we recall the definition of $\alpha _m$ as

4 $$\begin{aligned} \alpha _m = \arctan \frac{\omega h_0}{U} - \theta _0 \approx \frac{\omega h_0}{U} - \theta _0 \end{aligned}$$

where the approximation holds for small values of the pure heave incidence angle ${\omega h_0}/{U}$. If this is the case, the following simple relation between $\alpha _m$ and $\Theta$ holds

5 $$\begin{aligned} \frac{\alpha _m}{\theta _0} = \frac{1 - \Theta }{\Theta } \end{aligned}$$

whose physical meaning is very clear: as the feathering parameter is tending to one, i.e. the locomotion velocity is approaching the phase velocity *c*, the value of $\alpha _m$ tends to zero. From Fig. 4b we may easily deduce that the maximum propulsor efficiency occurs for very large values of $\alpha _m$ while the minimum of the cost of transport for the whole body occurs for large values of $\Theta$. The corresponding values of the parameters $\alpha _m$ and $\Theta$ are well reproducing results proposed in the literature for the search of optimal performance conditions. Namely, several findings confirm that fish select cruising speed usually very close to the phase velocity (i.e. $\Theta$ within $0.7-0.8$) when they have to cover large distances^26,27^. On the opposite, large values of the angle of attack, within $15^{\circ }-25^{\circ }$, are associated with higher propulsor efficiency^5^ and are more favorable when a large locomotion speed is required for escape-like gaits.

## Final remarks

When studying flapping airfoils under an incoming uniform flow, the focus is usually on the ability of the propulsor to generate a large thrust to balance the resistance, together with a high propulsive efficiency. Obviously, if the thrust of the propulsor prevails over the body drag an acceleration follows leading to different operating conditions. Once a certain body has been selected, a constant drag coefficient is prescribed hence it is more comfortable to adopt a self-propulsion approach which, by assuring the force balance, leads to a straightforward evaluation of the energy consumption together with the proper locomotion speed. In this way, we recover the procedure usually adopted in undulatory swimming governed by the phase velocity of a traveling wave. This choice is encouraged by the analogy illustrated before which introduces an asymptotic velocity also in the case of oscillatory swimming. The presence of a virtual body with its prescribed resistance allows, in the frame of a self-propulsion procedure, to evaluate also a well-defined propulsor efficiency to be contrasted with the cost of transport of the whole body. The results clearly indicate two different optimal swimming conditions: the first, characterized by a large locomotion velocity and a large angle of attack, is associated with the maximum propulsive efficiency; the second, associated with the minimum cost of transport, is characterized by a lower locomotion velocity and a quite large value of the feathering parameter. The contemporary observation of these different measures and the understanding of their validity for different swimming demands overcomes the conflicting opinions appearing in the literature about the best procedure to evaluate swimming performance^39^. In line with the overall discussion, we support here the use of a self-propulsion approach for the study of oscillatory swimming to obtain a direct evaluation of the performance, either for cruising long-range motions or for fast escape-like gaits. As a further point, the self-propelled axial motion is propaedeutic for the extension to lateral and angular degrees of freedom which drive the performance^28^ and better represents the swimming gaits observed in nature. To this purpose, the study of a swimming body under a prescribed uniform flow is not suitable, since no recoil motion may be accounted for, and the fully free locomotion is confirmed as the natural approach to obtain meaningful results.

## Materials and methods

The self-propelled axial motion of a swimming body with velocity ${\mathbf {u}}_b$ is analyzed by considering a two-dimensional body ${{{\mathscr {B}}}}$ within an unbounded fluid domain ${{{\mathscr {V}}}_\infty }$. No external forces are applied, hence only internal actions are exchanged between the deformable body and the surrounding fluid, otherwise quiescent. To the purpose, we adopt the classical impulse formulation^40,41^ for the linear fluid momentum which is expressed by two terms representing the field vorticity $\varvec{\omega }$ and the vortex sheet over the body surface as

6 $$\begin{aligned} {\mathbf {p}} = \int _{V_\infty } \rho \,{{\mathbf {x}}} \times \varvec{\omega }dV\ +\int _{\partial {\mathscr {B}}} \rho \,{{\mathbf {x}}}\times (\mathbf {n\times u^+})dS \end{aligned}$$

where $\varvec{n}$, the normal to the body surface $\partial {\mathscr {B}}$, points into the fluid domain ${V_{\infty }}$ and $\rho$ is the fluid density. A Helmholtz decomposition may be now applied to express the velocity field as the sum of the acyclic and vorticity related components:

7 $$\begin{aligned} \mathbf {u^+} = \mathbf \nabla \phi + \nabla \times \varvec{\Psi } = \mathbf \nabla \phi + {\mathbf {u}}_w \end{aligned}$$

where $\phi$ and $\varvec{\Psi }$ are referred to as the scalar and the (solenoidal) vector potential, and are given by the solution of the Laplace/Poisson equation, subject to the impermeable boundary condition on $\partial {\mathscr {B}}$, i.e. $\varvec{\nabla }\phi \cdot \varvec{n} = \varvec{u}_b \cdot \varvec{n}$ and $(\varvec{\nabla }\times \varvec{\Psi })\cdot \varvec{n} = 0$ respectively, and to the vanishing velocity at infinity. It follows that the total impulse $\varvec{p}$, which does not suffer the poor convergence of the momentum over an unbounded domain^42,43^ and whose time derivative gives the forces exchanged between the body and the surrounding fluid, may be expressed as the sum of the potential and vortical impulses, $\varvec{p}_{\phi }$ and $\varvec{p}_{v}$, as

8 $$\begin{aligned} {\mathbf {p}}_\phi = - \rho \; \int _{\partial {\mathscr {B}}} \phi \, {{\mathbf {n}}} \, dS \quad {\mathbf {p}}_v = \int _{V_{\infty }}\rho \; {{\mathbf {x}}} \times \varvec{\omega }dV\ + \int _{\partial {\mathscr {B}}}\rho \; {{\mathbf {x}}}\times ({\mathbf {n}} \times {\mathbf {u}}_w) \; dS \end{aligned}$$

where the bound vorticity due to ${\mathbf {u}}_w$, properly added to the released vorticity $\varvec{\omega }$, gives the additional vorticity introduced by Lighthill. The mathematical model, described in detail for undulatory free swimming in Paniccia et al.^31^, has been partially reported in the Supplementary Material and properly reshaped for the axial oscillatory swimming given by a flapping foil in the presence of a virtual body. The flow solutions are obtained by a simple inviscid procedure easily extendable to a classical vortex method by introducing the diffusion of the vorticity as detailed in a previous paper^44^. In the present work, a standard viscous solver, validated against the one used by Lin et al.^25^, has been used to provide a comparison of the results and an overall assessment of the inviscid procedure.

The sinusoidal heave and pitch motions with amplitudes $h_0$ and $\theta _0$, respectively, are characterized by an angular oscillation frequency $\omega = 10\pi ~rad/s$ and are separated in phase by an angle $\phi = \pi /2$ (pitch leading). The ratio $A_{h}/A_{TE}$ between the non-dimensional peak-to-peak trailing edge amplitude for pure heave motion and for combined heave and pitch motions is varied in the range $0.4\sim 0.98$ and the pitch oscillation amplitude $\theta _0$ follows to maintain the prescribed $A_{TE}$. Finally, for the virtual body, the drag coefficient $C_D$ is set equal to 0.25 and the mass *m* of the total body, i.e. virtual body plus propulsor, is set equal to 4.5 *kg*. For the details about the numerical procedures and choice of the parameters, see the Supplementary Material.

## Supplementary information

Supplementary Information.

Supplementary Video 1.
