# Supplementary material for: The performance of a flapping foil for a self-propelled fishlike body
Source: Sci Rep. 2021 Nov 16;11:22297. doi: 10.1038/s41598-021-01730-4 (PMC8595632; doi:10.1038/s41598-021-01730-4)
Supplement: Supplementary file 1 — Supplementary Information. [file 41598_2021_1730_MOESM1_ESM.pdf]

SUPPLEMENTARY MATERIAL - The performance of a flapping foil for a self-propelled fishlike body.

Damiano Paniccia[1,*,+], **Luca Padovani**[1,+], **Giorgio Graziani**[1,+], **and Renzo Piva**[1,+]

[1]Dept. of Mechanical and Aerospace Engineering, Univ. of Rome "La Sapienza", Rome, Italy
[*]damiano.paniccia@uniroma1.it
[+]these authors contributed equally to this work

1 The model for axial motion

As illustrated in the main text, we intend to study the axial motion of a swimming body $\mathscr{B}$ which is moving with a velocity $\mathbf{u}_b$ within an unbounded fluid domain $\mathscr{V}_\infty$. To this purpose we assume an unbounded $2D$ incompressible flow field, with constant density ρ, whose velocity vanishes at the far field boundary. We report below the main steps of the procedure to obtain the final equation to be solved for the numerical results.
The locomotion is obtained by coupling the body dynamics and the actions exchanged with the fluid. If we consider the body-fluid system ($\mathscr{B}+\mathscr{V}_\infty$), no external forces are present and therefore the linear momentum is conserved:

$$\frac{d}{dt}\int_{\mathscr{B}} \rho_b\,\mathbf{u}_b\,dV + \frac{d\mathbf{p}}{dt} = 0 \tag{S1}$$

where the time derivative of the total impulse $\boldsymbol{p}$ gives the force acting on the body and ρ_b is the body density. Let us now divide the whole body into an active part $\mathscr{B}_T$ given by the tail and a completely passive one, named virtual body $\mathscr{B}_V$, whose presence is attested only by its mass and its viscous drag in the axial direction. By using a Cartesian frame of reference $(\mathbf{e}_1, \mathbf{e}_2, \mathbf{e}_3)$ and by isolating the unknown locomotion speed $\mathbf{u}_0 = U\,\mathbf{e}_1$, the total motion of the entire body may be split into

$$\mathbf{u}_b = \begin{cases} \mathbf{u}_0 + \overline{\mathbf{u}} & \text{if } x \in \mathscr{B}_V \\ \mathbf{u}_0 + \mathbf{u}_T & \text{if } x \in \mathscr{B}_T \end{cases} \tag{S2}$$

where $\mathbf{u}_T$ is given by the prescribed heave and pitch motion of the tail

$$\mathbf{u}_T = V\,\mathbf{e}_2 + (\mathbf{x}-\mathbf{x}_0)\times\Omega\,\mathbf{e}_3 \qquad \mathbf{x} \in \mathscr{B}_T \tag{S3}$$

where $\mathbf{x}_0$ is the position of the pivot point and V and Ω are the lateral and angular heave and pitch velocity, respectively.
Since "... *the fish's muscular contractions can only determine changes in its shape relative to the centre of gravity.*" (Lighthill '70[1]), the velocity of the virtual body $\overline{\mathbf{u}}$ is taken to satisfy the conservation of linear and angular momenta for the entire body system, including the prescribed movement of the tail.
By combining (S1) and (S2) we obtain

$$\frac{d}{dt}(m_b\,\mathbf{u}_0) + \frac{d\mathbf{p}}{dt} = 0 \tag{S4}$$

The surface integrals appearing within the total impulse $\mathbf{p}$ may be decoupled into the contribution from the tail and that from the virtual body. By taking the component of (S4) along $\mathbf{e}_1$ to solve for the locomotion along the axial direction, we assume the virtual body contribution to be represented by its overall resistance D, leading to

$$\frac{d}{dt}(m_b U) + \frac{dp}{dt} + D = 0 \tag{S5}$$

where the axial component p of the impulse contains the contribution from the tail. By assuming zero initial conditions, (S5) gives:

$$m_b U + p = -\int_0^t D\,dt \tag{S6}$$

The scalar potential introduced by the Helmholtz decomposition is evaluated according to the related boundary conditions on the tail boundary

$$\frac{\partial \phi}{\partial n} = \boldsymbol{u}_b \cdot \boldsymbol{n}\Big|_T$$

Finally, the potential impulse may be expressed in terms of the added mass coefficients introduced in the classical treatises (see e.g.[2]) that, for completeness, are reported below. For a foil motion with unknown axial velocity U and prescribed lateral and angular velocity V and Ω respectively, we consider the Kirchhoff base potentials Φ_1, Φ_2 and Φ_3 defined through the boundary conditions

$$\frac{\partial \Phi_1}{\partial n} = \mathbf{n} \cdot \mathbf{e}_1 \qquad \frac{\partial \Phi_2}{\partial n} = \mathbf{n} \cdot \mathbf{e}_2 \qquad \frac{\partial \Phi_3}{\partial n} = (\mathbf{x} - \mathbf{x}_0) \times \mathbf{n} \cdot \mathbf{e}_3 \tag{S7}$$

to have $\phi = U\Phi_1 + V\Phi_2 + \Omega\Phi_3$. It follows for the added mass coefficients in the axial direction m_{1j} the expression

$$m_{1j} = \rho \int_{\partial \mathscr{B}_T} \frac{\partial \Phi_1}{\partial n} \Phi_j \, dS \tag{S8}$$

The prescribed lateral and angular tail velocities within p_ϕ, which are multiplied by m_{12} and m_{13} respectively, can be shifted to the r.h.s. to yield the equation for the axial body motion:

$$U\,(m_{11} - m_b) = -V\,m_{12} - \Omega\,m_{13} + p_v + \int_0^t D\,dt \tag{S9}$$

The drag term appearing on the r.h.s. of (S9) is expressed as $D = \frac{1}{2}\rho U^2 L C_D$, where L is the body length and C_D is the prescribed drag coefficient. The numerical solution of the equation, quite trivial at steady state, requires a simple numerical treatment to manage the transient phase of the locomotion velocity.
As a final remark, the input power P is evaluated as

$$P = f \int_{t+1/f} (LV + M\Omega)\, dt \tag{S10}$$

where the lift is $L = \frac{d\boldsymbol{p}}{dt} \cdot \boldsymbol{e}_2$ and the moment is $M = \frac{d\boldsymbol{\pi}}{dt} \cdot \boldsymbol{e}_3$ with the angular impulse about the tail leading edge defined as

$$\boldsymbol{\pi} = -\frac{1}{2}\left[\int_{V_\infty} \rho\,|x|^2 \boldsymbol{\omega}\, dV + \int_{\partial \mathscr{B}_T} \rho\,|x|^2 (\boldsymbol{n} \times \boldsymbol{u}^+)\, dS\right] \tag{S11}$$

2 Techniques and data for the simulations

A flapping foil acting as the propulsor of a fishlike body has been studied by a well-known inviscid numerical procedure with the aim to suggest a neat and simple way to investigate the performance of oscillatory swimming fish. The flow solutions about the flapping airfoil are obtained by a potential code based on Hess and Smith[3] approach together with a suitable unsteady Kutta condition and a proper evolution of the wake behind the airfoil as indicated by Basu and Hancock[4]. Finally, the locomotion of the whole-body (airfoil + virtual body) is obtained by satisfying the conservation of total momentum in the forward direction. Further details on the adopted methodology can be found in Paniccia et al.[5], where also lateral and angular directions are considered.

With regard to the viscous results reported in the manuscript, the flow solutions have been obtained by using a CFD solver for

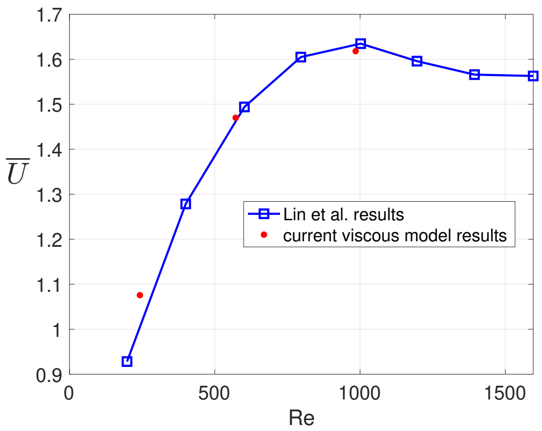

Figure S1. Comparison between the current viscous model results and the ones by Lin et al.[8] for a self-propelled heaving and pitching foil.

the Navier-Stokes equations based on an immersed boundary method. The numerical code has been developed by Popinet[6] and it was already successfully adopted in the field of self-propelled fish locomotion by[7] among others. The forward locomotion of the whole-body follows from the evaluation of the axial force exerted by the surrounding fluid and from the solution of the Newton's second law in the axial direction only. The current viscous solver results are validated against the ones obtained by Lin et al.[8] for the same heaving and pitching conditions as shown in fig.S1.

Let us now describe in more details the parameters we selected for the flapping foil and for the virtual body in front of it. We assume the body length $L = 1\ m$ and the tail length l, here taken as the reference length, equal to $1/7\ L$, ratio frequently observed in nature for real tuna. The presence of the virtual body is only attested by its mass m_b and its resistance in terms of a prescribed drag coefficient C_D. The values of m_b is based on a NACA0018 airfoil geometry with chord length equal to $6l$ and unit density, i.e. $m_b \approx 4.4\ kg$, and the value of $C_D \approx 0.25$ has been selected as the mean value of the experimental data by White et al.[9] for their robotic tuna. Finally, the flapping foil representing the tail is modeled as a NACA0012 airfoil of chord length l with mass m_t equal to $0.08\ kg$, leading to a total mass of the whole-body $m = m_b + m_t \approx 4.5\ kg$. For further geometrical details, fig.S2 reports the sketch of the tail from the inset of fig.1 in the main text where the whole-body is shown.

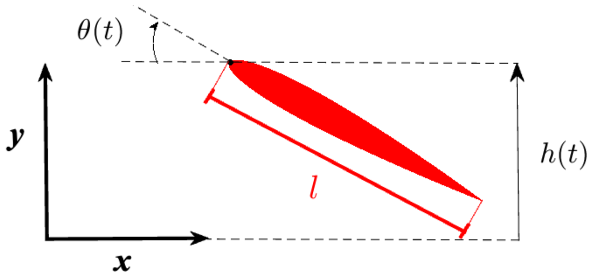

Figure S2. Sketch of the tail from the inset of fig.(1) in the main text.

The heave motion of the tail is defined as

$$h(t) = h_0 \sin(\omega t) \tag{S12}$$

where h_0 is the heave amplitude and ω is the oscillation angular frequency which is equal to $10\pi\ rad/s$ in the present case. The pitch motion is defined as

$$\theta(t) = \theta_0 \sin(\omega t + \phi) \tag{S13}$$

where θ_0 is the pitch amplitude and the phase angle ϕ is equal to $\pi/2$. For pitch motions about the leading edge and for sufficiently small θ_0 it is possible to approximate the value of the non-dimensional peak-to-peak trailing edge amplitude with the following analytical expression (see also[10])

$$A_{TE} = \sqrt{(2\theta_0)^2 + A_h^2} \tag{S14}$$

where $A_h = 2h_0/l$ is the non-dimensional amplitude for a pure heave motion. Two different trailing edge amplitudes have been considered, namely $A_{TE} = 1.0$ and $A_{TE} = 1.5$, for the ratio A_h/A_{TE} ranging within $0.4 \sim 0.98$ while the pitch amplitude θ_0 follows directly from (S14). For the sake of completeness, we summarize in Tab.S1 below all the input data used in our study.

A_{TE}=1.0			A_{TE}=1.5		
$\frac{h_0}{l}$	$\frac{A_h}{A_{TE}}$	θ_0	$\frac{h_0}{l}$	$\frac{A_h}{A_{TE}}$	θ_0
0.2000	0.4000	0.4583	0.3000	0.4000	0.6874
0.2500	0.5000	0.4330	0.3750	0.5000	0.6495
0.3000	0.6000	0.4000	0.4500	0.6000	0.6000
0.3500	0.7000	0.3571	0.5250	0.7000	0.5356
0.4000	0.8000	0.3000	0.6000	0.8000	0.4500
0.4300	0.8600	0.2551	0.6450	0.8600	0.3827
0.4500	0.9000	0.2179	0.6750	0.9000	0.3269
0.4700	0.9400	0.1706	0.7050	0.9400	0.2559
0.4900	0.9800	0.0995	0.7350	0.9800	0.1492

Table S1. Values of $\frac{h_0}{l}$, $\frac{A_h}{A_{TE}}$ and θ_0 for A_{TE}=1.0 (left) and A_{TE}=1.5 (right).

3 The effect of forward oscillations

To proof that the oscillations in the forward velocity give a negligible contribution on large scale parameters like the cost of transport and the locomotion velocity we should realize a self-propelled motion with an axial velocity constrained to be perfectly constant, i.e. without the implicit oscillations. However, since the axial velocity is the unknown of the problem, we cannot make any constraint on this variable as we may do with the lateral and angular motions where it is quite easy to annihilate the values of the corresponding velocities (see[11]). To overcome this conundrum we report here for comparison the results obtained by a self propelled approach and the ones obtained by the prescribed uniform stream. Figure (S3a) shows the forward velocity obtained by the self-propelled fishlike body for $h_0 = 0.6$ and $A_{TE} = 1.5$. To compare this self-propelled case against an axial location fixed swimming case, we selected the mean forward velocity reached at steady state of the first case as the prescribed constant speed for the second one. It follows the same drag coefficient in both cases within the approximation obtained for the other variables. In fig.(S3b) we report the input power time evolution in one oscillation period for both the self-propelled and the fixed swimming cases. From the comparison we may appreciate a very small difference, less than 2%, between the two mean values that, in a first approximation, is quite negligible. This fact, explains why for the axial swimming, and exclusively in this case, each single result in one point of the parameter space may be indifferently obtained by the two mentioned approaches. Obviously, this is not true anymore for fully free swimming in presence of all the recoil motion components.
As an ultimate comment, when using the one or the other approach to explore the parameter space, the routes to find the optimal swimming performance are completely different. In fact, by taking the flapping foil data as a running parameter for the analysis, we may fix either the velocity, as in the prescribed stream approach, or the body drag coefficient, as in the self propulsion approach and this last procedure, in our opinion, is certainly preferable since the aim is to find these results for a certain fishlike body.

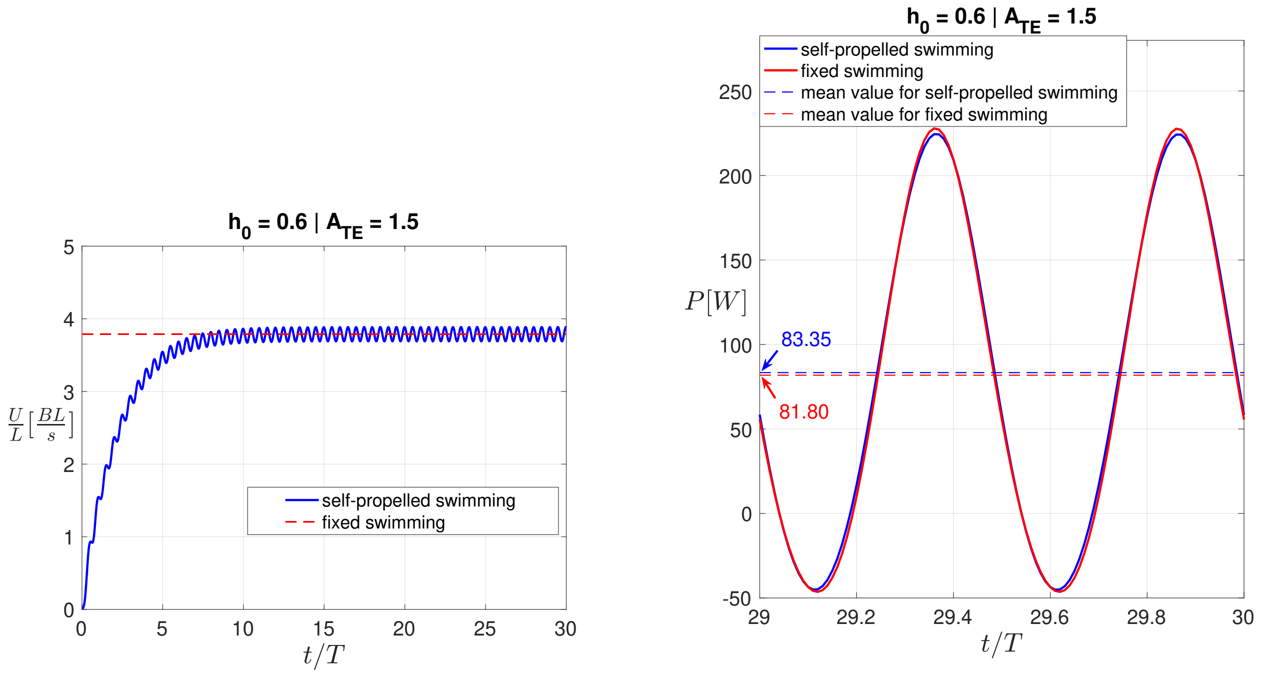

(a) **(b)**

Figure S3. (S3a) Forward velocity for the self-propelled swimming case. The steady state mean value has been selected as the prescribed value for the fixed swimming case. (S3b) Comparison between the input power for the self-propelled swimming case and for the fixed swimming case. The two different mean values are reported in blue and red respectively.

Supplementary Video Legend

Animation of the swimming fishlike model and the related vortex wake. The appearing deformation of the virtual body (gray) is not effective, but instrumental to make a smooth and nice connection with the oscillating tail (red).

References

1. Lighthill, J. Aquatic animal propulsion of high hydromechanical efficiency. *J. Fluid Mech.* **44**, 265–301 (1970).
2. Lamb, H. *Hydrodynamics* (Cambridge Univ. Press, 1975), 6 edn.
3. Hess, J. L. & Smith, A. M. O. Calculation of potential flow about arbitrary bodies. *Prog. Aerosp. Sci.* **8**, 1–138 (1967).
4. Basu, B. C. & Hancock, G. J. The unsteady motion of a two-dimensional aerofoil in incompressible inviscid flow. *J. Fluid Mech.* **87**, 159–178 (1978).
5. Paniccia, D., Graziani, G., Lugni, C. & Piva, R. On the role of added mass and vorticity release for self propelled aquatic locomotion. *J. Fluid Mech.* **918**, A45, DOI: 10.1017/jfm.2021.375 (2021).
6. Popinet, S. Gerris: a tree-based adaptive solver for the incompressible euler equations in complex geometries. *J. Comput. Phys.* **190**, 572–600 (2003).
7. Wang, L. & Wu, C. J. An adaptive version of ghost-cell immersed boundary method for incompressible flows with complex stationary and moving boundaries. *Sci. China* **53**, 923–932 (2010).
8. Lin, X., Wu, J. & Zhang, T. Performance investigation of a self-propelled foil with combined oscillating motion in stationary fluid. *Ocean. Engin.* **174**, 33–49 (2019).
9. White, C. H., Lauder, G. V. & Bart-Smith, H. Tunabot flex: a tuna-inspired robot with body flexibility improves high-performance swimming. *Bioinspir. Biomim.* **16**, 026019 (2021).
10. Young, J. *Numerical simulation of the unsteady aerodynamics of flapping airfoils*. Ph.D. thesis, School of Aerosp. Civil and Mech. Engin. Univ. New South Wales, AUS (2005).

11. Paniccia, D., Graziani, G., Lugni, C. & Piva, R. The relevance of recoil and free swimming in aquatic locomotion. *J. Fluids Struct.* **103**, 103290, DOI: 10.1016/j.jfluidstructs.2021.103290 (2021).
